# Proteome changes induced by *Pyrenophora tritici-repentis* ToxA in both insensitive and sensitive wheat indicate senescence-like signaling

**DOI:** 10.1186/s12953-014-0060-3

**Published:** 2015-02-05

**Authors:** Jacqueline Day, Roman Daniel Gietz, Christof Rampitsch

**Affiliations:** Agriculture and Agrifood Canada, 100 Route 101, Morden, MB Canada; Department of Biochemistry and Medical Genetics, University of Manitoba, Winnipeg, MB Canada

**Keywords:** Ptr ToxA, Proteomics, Tan spot

## Abstract

**Background:**

*Pyrenophora tritici-repentis* is a phytopathogenic fungus which causes tan spot on wheat. Some races of *P. tritici-repentis* produce host-specific toxins which present symptoms of chlorosis or necrosis on susceptible wheat cultivars. One such toxin is Ptr ToxA, which enters mesophyll cells through a putative toxin-receptor and localizes with chloroplasts, ultimately causing damage and necrosis on leaves. These symptoms can occur even in the absence of the pathogen. Insensitive cultivars lack the receptor and Ptr ToxA cannot enter cells. The molecular mechanisms surrounding this plant-pathogen interaction are still largely unknown, although some details have begun to emerge.

**Results:**

Using 2-D electrophoresis, fifteen protein changes were identified reproducibly in the leaf proteomes of a sensitive and an insensitive cultivar over three days after inoculation of purified Ptr ToxA. Functional analysis of the proteins indicated that senescence signals may be induced in the sensitive cultivar. In the insensitive cultivar proteins involved in some features of senescence inhibition were seen. Complementary responses at the biochemical level may be actively promoting a localized senescence-like response in sensitive wheat cultivars whilst actively inhibiting this response in insensitive cultivars.

**Conclusion:**

This is the first report of a biochemical response in an insensitive cultivar in this plant-pathogen interaction. Findings support the involvement of ethylene, and the activation of complementary pathways in sensitive versus insensitive wheat cultivars responding to Ptr ToxA. The nature of the system permits using purified toxin to mimic disease, which eliminates the pathogen proteome and ensures a synchronous response in inoculated leaves.

**Electronic supplementary material:**

The online version of this article (doi:10.1186/s12953-014-0060-3) contains supplementary material, which is available to authorized users.

## Background

Ptr ToxA is a proteinaceous host-specific toxin produced by some races of *Pyrenophora tritici-repentis* (Died.) Drechs. (anamorph: *Drechslera tritici-repentis* (Died.) Shoem.), the casual agent of tan spot, a foliar disease of wheat (*Triticum aestivum* L.). In sensitive cultivars administration of the toxin alone is sufficient to cause characteristic necrotic symptoms reproducibly [1]. Toxin sensitivity follows the toxin model, which is the inverse of the gene-for-gene model: the absence of necrosis (insensitivity) is the result of non-recognition, while the presence of necrosis (sensitivity) is due to host-recognition of the toxin by the corresponding host-gene product [2]. This host-gene product is encoded by a single dominant gene, *Tsn1*, located on chromosome 5BL [3]. Chu *et al*. [4] have speculated that recognition of ToxA by Tsn1 leads to downstream signalling even though Ptr ToxA itself does not directly interact with Tsn1. Although Tsn1 confers sensitivity, it contains domains such as S/TPK and NBS-LRR, which are common features of resistance genes [4].

Ptr ToxA has a unique structure and sequence, within which is a conserved Arg-Gly-Asp (RGD) domain [5]. This domain is crucial for toxin activity. A mutation in this domain to an RAD or RGE results in an inability to induce necrosis [6]. Infiltration of Ptr ToxA with an RGD motif-containing peptide reduces the toxin’s effect by more than 60% within the first 3.5 hours, suggesting that these peptides are competing with Ptr ToxA for putative binding sites [6]. The RGD domain is used by adenoviruses to gain entry into a cell [7] and by intergrins to bind receptors that activate signalling cascades [8]; both are features shared by Ptr ToxA.

In sensitive cultivars the toxin is internalized [9]; conversely in insensitive cultivars the toxin is unable to cross from the apoplastic space into the mesophyll cells. However, forcing toxin entry into leaf cells through ballistics results in toxicity to both insensitive and sensitive cultivars demonstrating that internalization is required for sensitivity [9].

A significant quantity of ROS are released by 18 hours post infiltration (hpi) in sensitive cultivars [10]. The source of these ROS remains unknown but may be due to a functional disruption of ToxA BP1. Using yeast two hybrid analysis Manning and colleagues showed that Ptr ToxA binds to ToxABP1, a PSII protein [11]. They speculated that this binding may disrupt the electron transport chain and release ROS. A similar disruption also occurred in ToxABP1 knockouts [12,13]. It is also possible that the production of ROS is a result of transcriptional changes induced by Ptr ToxA in sensitive cultivars since transcriptional changes occur as early as 3 hpi, well in advance of the significant increase of ROS at 18 hpi [14]. Photosystems I and II along with other photosynthetic proteins exhibited decreased regeneration efficiencies from 9 hpi to 14 hpi due to transcriptional repression [14]. Transcripts of genes involved in ethylene biosynthesis were also elevated after Ptr ToxA infiltration [14]. Increased levels of ethylene could account for the large quantity of ROS seen at 18 hpi since ethylene is involved in the production of superoxide [15].

Regardless of the origin of these ROS, the evidence that this is the key factor in inducing necrosis is strong. Plants injected with ROS inhibitors showed reduced necrosis and plants placed in darkness to reduce ROS production presented less necrosis, implying that the onset of necrosis is dependent on ROS quantity [10]. In sensitive leaves, ROS are detected at 9 hpi and quickly increased to a large quantity at 18 hpi – the point at which necrosis is visible macroscopically [10]. Interestingly, insensitive leaves also produce a detectable supply of ROS starting as early as 6 hpi and they continue to be detectable at this level up to 24 hpi, however the quantity is significantly lower than in sensitive leaves, and no visible necrosis results, implying that ROS threshold for necrosis may exist.

Adhikari and colleagues also reported measurable differences in the insensitive line between buffer-treated and toxin-treated leaves [16]: Ptr ToxA produced a one-fold increase in cell death over control at 12 hpi, but this rapidly decreased to less than 25% by 48 hpi in insensitive while the sensitive cultivar resulted in a two fold increase in cell death over the control from 24 hpi to 48 hpi when treated with Ptr ToxA [16]. These two studies both suggest that insensitive cultivars also react to the toxin albeit at a lower level. For this reason a Ptr ToxA insensitive cultivar was included in the present study even though most studies have focused only on molecular changes within sensitive wheat lines.

Within Ptr ToxA sensitive leaves Ca^2+^ mediated signalling is required to induce necrosis [17,18] and transcripts of calcium kinase and other related calcium transcripts are detected as early as 3 hpi [14]. Defence genes such as those involved in lignification, PR proteins and proteins involved in SA, JA and ethylene biosynthesis were also found to be upregulated. A general decrease of transcripts involved in photosynthesis and transcription occurred during 9–14 hpi.

Deciphering the molecular mechanisms of the host response to Ptr ToxA for both insensitive and sensitive cultivars through proteomics will aid in identifying new proteins and indicate if up-regulation or down-regulation of transcripts leads to changes in protein abundance. Here we present a comparative proteomics study to identify changes in each cultivar infiltrated with buffer in the presence or absence of Ptr ToxA at day 1, day 2 and day 3. Changes in the proteomes of both sensitive and insensitive wheat cultivars suggest that the two cultivars are reacting, possibly in an opposing manner. Many of the identified proteins in the sensitive cultivar suggest cellular shutdown, while the insensitive cultivar appears to be up-regulating key metabolic pathways. This is the first evidence of biochemical changes in the proteome of an insensitive wheat cultivar challenged with Ptr ToxA.

## Results

### *Pyrenophora tritici-repentis* ToxA isolation

**Figure 1:**
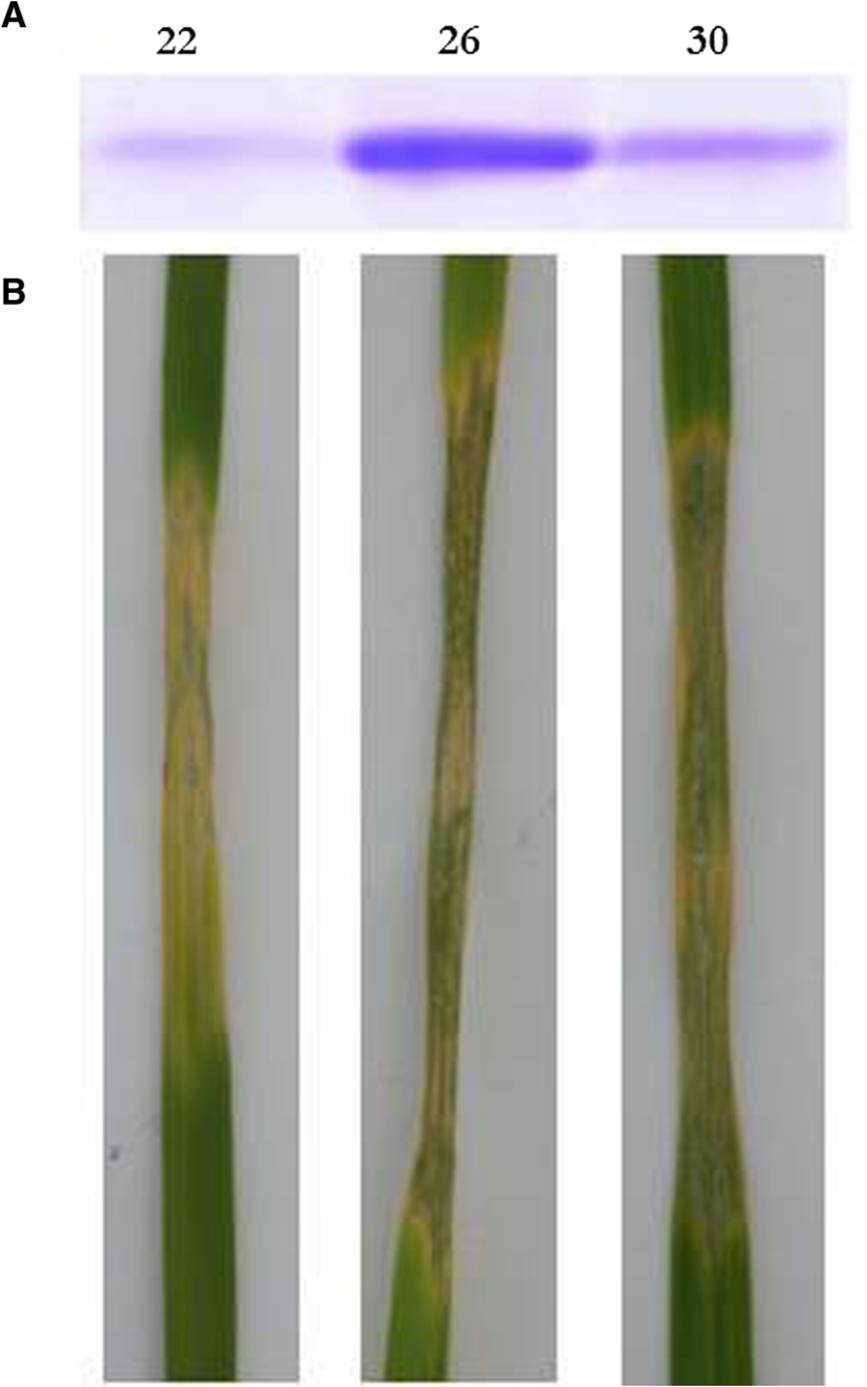
**Toxin isolation and infiltration. A:** selected fractions from CM cellulose column separated on 12.5% Tris-tricine SDS polyacrylamide gels and stained with Coomassie brilliant blue. **B:** corresponding fractions 5 days post infiltration in sensitive wheat leaves.

To locate Ptr ToxA in chromatograms, fractions were first infiltrated into leaves and then resolved on Tris-tricine electrophoresis gels to verify the presence of Ptr ToxA (Figure 1). A prominent band in each fraction was identified at the expected M_r_ of 13.9 kDa and verified by *de novo* sequencing. The intensity of each band corresponded qualitatively to the level of necrosis observed in the leaves.

### Changes in the wheat leaf proteome

**Figure 2:**
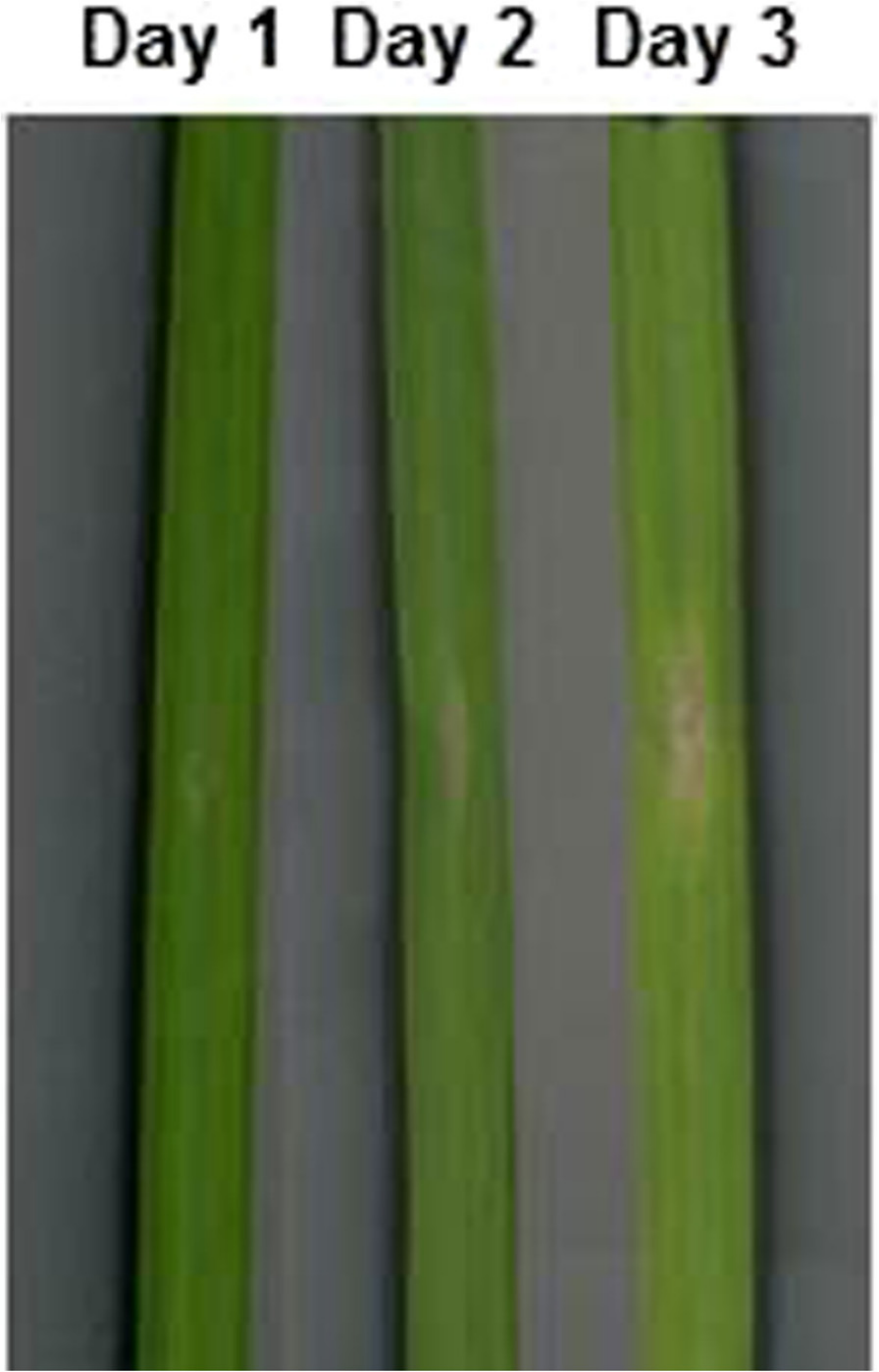
**Necrosis progression.** Wheat leaves were infiltrated with a 0.9 ng.μl^−1^ solution of Ptr ToxA and collected at day 1, day 2, and day 3 post infiltration.

**Figure 3:**
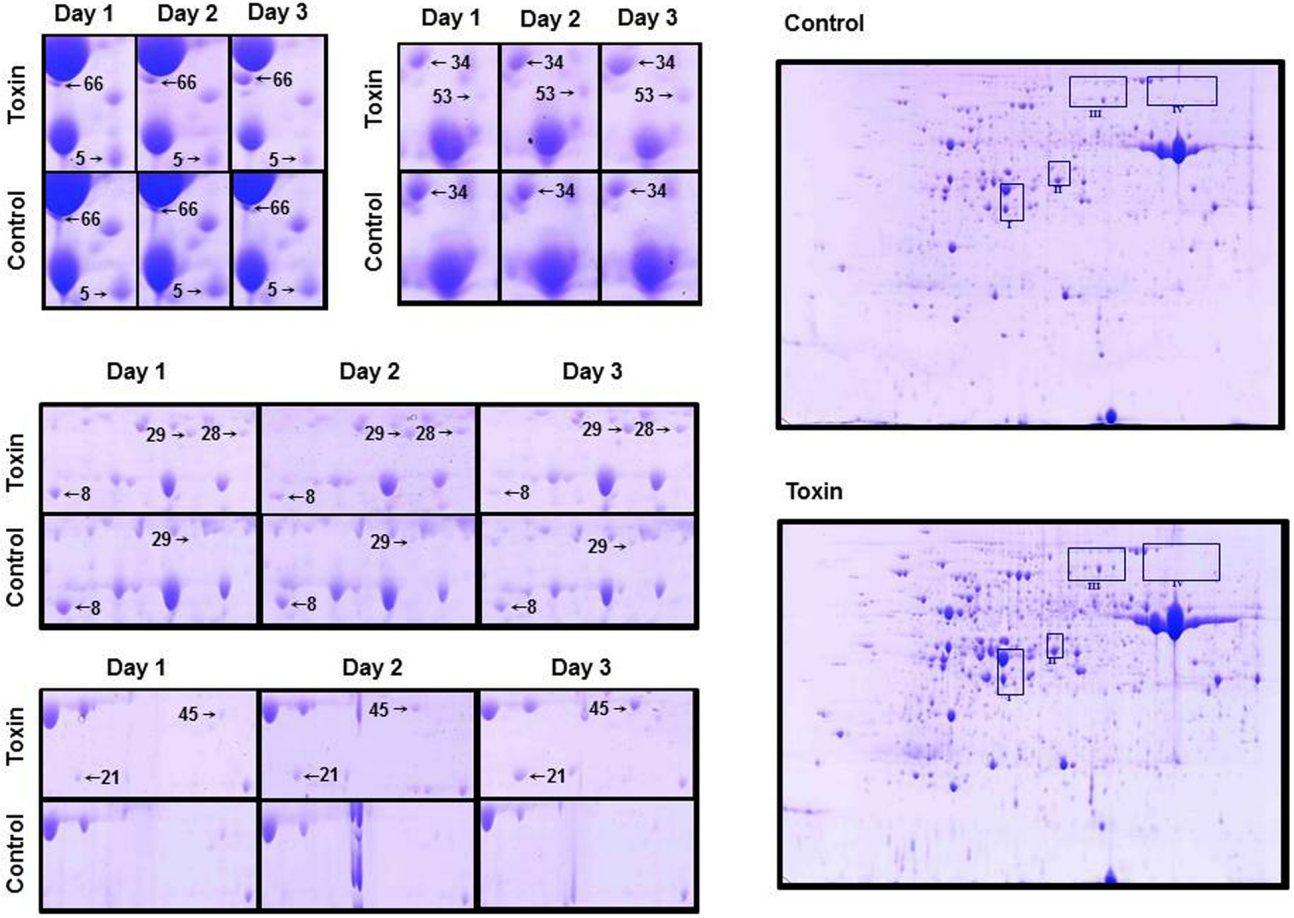
**Proteome changes in sensitive wheat leaves infiltrated with Ptr ToxA or control buffer.**

**Figure 4:**
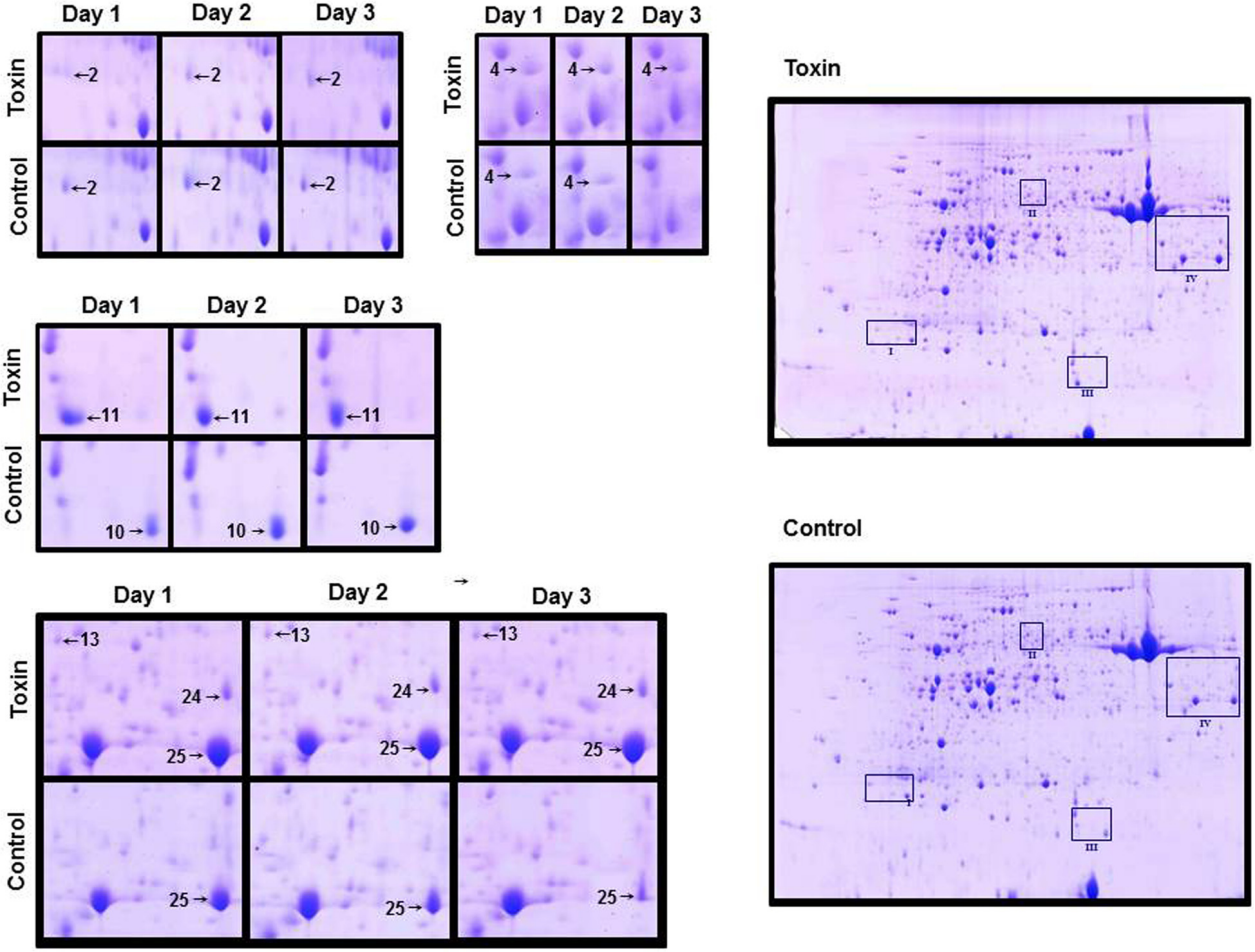
**Protein changes in 2D electrophoresis of Amazon wheat leaves infiltrated with Ptr ToxA or control buffer.**

Necrosis was only observed in toxin treated sensitive wheat leaves and increased progressively with time (Figure 2). The insensitive leaves and all control treatments remained asymptomatic. After separation of infected leaf proteomes by 2-D electrophoresis (IEF × SDS-PAGE), protein spots were selected for analysis only if changes were consistent in both biological replicates. The proteome changes were subtle: eight protein spots were selected from the susceptible wheat cultivar Glenlea (Figure 3) and seven from the resistant cultivar Amazon (Figure 4). Seven proteins were identified from Glenlea with spots 28 and 29 containing the same protein with the same M_r_ but differing in pI. From Amazon, a protein was also identified in two separate spots, thus six individual proteins were identified in this cultivar. Each identified protein had more than two significant peptide matches with total coverage ranging from 20–77% (Table 1, Glenlea & Table 2, Amazon) and all were mainly identified from homologous proteins matched in either *T. aestivum*, *Oryza sativa* or *Hordeum vulgare*. Calculations of qantitative values for each spot are shown in Additional file 1: Table S1.

**Table 1:** **Affected proteins tentatively identified in sensitive leaves infiltrated with Ptr ToxA**

**Spot** ^**(1)**^	**Putative Identity**	**Identification**	**Taxonomy**	**Sequence** ^**(2)**^	**Score** ^**(3)**^	**Ratio toxin:control** ^**(4)**^			**Trend**
						**Day 1**	**Day 2**	**Day 3**	
5	Ferredoxin-NADP(H) oxidoreductase	gi 20322473	*Triticum aestivum*	LYSIASSALGDFGDSK	114	0.485	0.368	0.295	Decrease
				DPNATIIMLATGTGIAPFR	111				
				TOTAL	1278				
8	putative GTP-binding protein typA	gi 50906979	*Oryza sativa*	INIIDTPGHSDFGGEVER	89	0.348	0.336	0.183	Decrease
				ALEFGHAVVVVVNK	107				
				TOTAL	784				
28	ATP-dependant Clp protease ATP-binding subunit precursor	gi 26518520	*Oryza sativa*	LDMSEYMER	75	4.470	35.726	8.728	Increase
				RKPFTVVLLDEIEK	81				
				TOTAL	732				
29	ATP-dependant Clp protease ATP-binding subunit precursor	gi 26518520	*Oryza sativa*	GELQCIAATTLDEHR	94	3.316	5.155	4.355	Increase
				GALDQFCLDLTTQASGGFIDPIIGREEEIER	100				
				TOTAL	731				
21	Methionine synthase 2 enzyme	gi 68655500	*Hordeum vulgare*	GMLTGPVTILNWSFVR	116	2.455	5.030	35.526	Increase
				ALAGQKDEAYFAANAAAAQASR	116				
				TOTAL	2165				
34	putative Ado Met synthase 3	gi 68655446	*Hordeum vulgare*	NIGFISDDVGLDADR	122	0.533	0.800	1.715	Increase
				ENFDFRPGMISINLDLKK	123				
				TOTAL	1475				
53	Cinnamyl alcohol dehydrogenase 2a	gi 15428280	*Festuca arundinacea*	LVLMGVIAEPLSFVSPMVMLGR	69	0.509	2.196	3.536	Increase
				IPAGLAPEQAAPLLCAGVTVYSPLK	85				
				TOTAL	633				
66	Glutamine synthase isoform GS1b	gi 71361902	*Triticum aestivum*	IIAEYIWIGGSGMDLR	100	0.964	0.929	3.011	Increase
				HETADINTFSWGVANR	100				
				TOTAL	1256				

^**(1)**^Spot numbers correspond to numbers in Figure 3.

^**(2)**^The two most significant peptide sequences are shown.

^**(3)**^Mascot Ions scores are −10.Log(P), where P is the probability that the observed match is a random event. Individual ions scores > 47 indicate identity or extensive homology (p < 0.05) [46].

^**(4)**^Quantitative values are explained in the text and in Additional file 1: Table S1.

**Table 2:** **Affected proteins tentatively identified in insensitive leaves infiltrated with Ptr ToxA**

**Spot** ^**(1)**^	**Putative Identification**	**Identity**	**Taxonomy**	**Sequence** ^**(2)**^	**Score** ^**(3)**^	**Ratio toxin:control** ^**(4)**^			**Trend**
						**Day 1**	**Day 2**	**Day 3**	
13	elongation factor 1 gamma-like protein	gi 29367403	*Oryza sativa*	NPLDLLPPSK	57	13.918	4.554	5.537	Decrease
				NFQMGVSNKTPEFLK	49				
				TOTAL	340				
2	Type III LHCII CAB precursor protein	gi 19023	*Hordeum vulgare*	WAMLGALGCVFPEVLQK	96	0.915	0.515	0.518	Decrease
				LAMFSMFGFFVQAIVTGK	55				
				TOTAL	365				
4	putative methylenetetrahydrofolate reductase	gi 50919385	*O. sativa*	SKAFPSLTYIAVNK	62	3.803	2.188	3.889	Increase
				QIGITCPIVPGIMPINNYK	49				
				TOTAL	354				
10	putative Rieske Fe-S precursor protein	gi 32394644	*Triticum aestivum*	GPAPLSLALVHADVDDGK	97	0.047	0.068	0.045	Only present in Control
				TLATYGINAVCTHLGCVVPWNAAENK	99				
				TOTAL	838				
11	putative Rieske Fe-S precursor protein	gi 32394644	*T. aestivum*	DKLGNDILVEDWLK	91	71.988	20.703	558.495	Absent in Control
				TLAQGLKGDPTYLVVESDK	89				
				TOTAL	674				
24	putative hydroxypyruvate reductase	gi 50904581	*O. sativa*	EGMATLAALNVLGK	78	8.351	6.485	3.270	Decrease
				EADVISLHPVLDK	82				
				TOTAL	711				
25	Glyceraldehyde-3-phosphate dehydrogenase A [2]	gi 120657	*Zea mays*	VPTPNVSVVDLVVQVSK	116	1.888	3.145	6.432	Increase
				YDSTLGIFDADVKPVGDNAISVDGK	86				
				TOTAL	1033				

^**(1)**^Spot numbers correspond to numbers in Figure 4.

^**(2)**^The two most significant peptide sequences are shown.

^**(3)**^Mascot Ions scores are −10.Log(P), where P is the probability that the observed match is a random event. Individual ions scores > 47 indicate identity or extensive homology (p < 0.05) [46].

^**(4)**^Quantitative values are explained in the text and in Additional file 1: Table S1.

In Glenlea, Clp protease ATP binding subunits were identified in two separate spots, one showing increased expression and the other appearing only in toxin infiltrated samples. Cinnamyl alcohol dehydrogenase, an enzyme involved in cell wall lignification and previously shown to be upregulated by *P. tritici-repentis* [19], was also seen to be up-expressed. The remaining proteins showing increased presence were methionine synthase, putative Ado Met synthase 3, and glutamine synthase. Only two proteins showed decreased abundance, ferredoxin-NADP oxidoreductase, an important component of photosynthesis, and putative GTP-binding protein, typA, which plays a role in oxidative stress.

In Amazon wheat, proteins involved in protein synthesis, methylenehydrofolate reductase and elongation factor 1, showed an increased abundance. Rieske Fe-S was identified in two separate spots, 10 and 11, however the spot with the lower pI was found only in the toxin induced sample while the isoform with a higher pI was found only in the control treated plant. Only one protein presented decreased abundance, LHC II CAB while the other photosynthetic protein, glyceraldehyde 3 phosphate dehydrogenase showed a significant increase in toxin treated plants. Hydroxypyruvate reductase also showed a significant increase when treated with Ptr ToxA.

## Discussion

The Ptr ToxA-wheat interaction is an excellent model for proteomic analyses because while the toxin itself does not contribute to the proteome, it still elicits the same host response as the fungus, with the advantage that disease onset is more synchronous than when using live pathogen. In addition, Glenlea and Amazon are near isogenic lines of wheat, thus induced changes are likely the result of the host-toxin interaction. This maximizes the chances of detecting molecular events involved in both sensitive and insensitive cultivars. Furthermore, stringent criteria were used to define protein changes: only proteins showing clear visual changes in all time points in two biological replicates were selected from 2-DE gels. Three of the eight proteins identified had been previously identified in transcriptional studies [14], however in many cases the proteins were related and appear to be involved in similar pathways in this plant-pathogen interaction.

### Sensitive response

Previous studies have indicated that Ptr ToxA affects photosynthesis by altering the homeostasis of PS I [10]. In the present study it is evident that PS I is also disrupted as demonstrated by the progressive decrease of ferredoxin-NADP(H) oxidoreductase (FNR), spot 5. FNR is required to create NADPH for the Calvin cycle and a lack of FNR decreases carbon fixation suggesting a decrease in nutrient source [20].

A feature of Ptr ToxA infiltration is cell wall lignification in sensitive cultivars [19]. This is supported by transcriptional studies which show cinnamyl alcohol dehydrogenase and caffeoyl-CoA-*O*-methyltransferase induction, both of which contribute to lignin biosynthesis [21].

Glutamine synthase GS1b, spot 66, a protein with multiple roles, showed a progressive increase throughout day 1 to day 3. Its role here may be to reassimilate ammonium released in the biosynthesis of lignin [22] but it has also been observed to be induced after biotic and abiotic stress [23,24]. GS1b is also a key indicator of senescence because of its involvement in nitrogen mobilization [25]. Other proteins identified in this study may also be involved in senescence pathways.

Protease induction occurs during the first stages of senescence for selective degradation to help drive the unfolding of specific proteins in preparation for lysis [26]. Two ATP-dependent caseinolytic protease (Clp) ATP-binding subunit precursors, spots 28 and 29, increased in expression throughout the time course, suggesting that there was a total increase in Clp, since a functional Clp requires an ATP subunit along with a regulatory subunit. The specific targets of the regulatory subunits are yet to be discovered but it is evident that Clps are required in higher plants under both optimal and stress conditions [27]. Some Clps are induced as a result of stress and during the onset of senescence [28]. By 48 hpi necrotic lesions are visible, thus it is possible that Clps are active here in selective degradation, a characteristic of senescence [29,30].

An increase in *S*-adenosyl methionine (Ado Met) synthase, spot 34, and methionine synthase was evident throughout the time points. Methionine synthase is the terminal enzyme step of methionine biosynthesis. Over 80% of the methionine produced in plants is used to produce Ado Met through Ado Met synthase [31]. Ado Met is a major source of methyl groups for the biosynthesis of nicotiamines, phytosiderophores, polyamines and ethylene [32]. Although Ado Met is a widely used precursor molecule, it is most likely being used for ethylene biosynthesis. This is supported by evidence from transcriptional studies that showed increased transcripts of 1-aminocyclopropane-1-carboxylase oxidase, aminocyclopropane-1-carboxylase synthase (and S-adenosylmethionine synthase 1) as a result of toxin infiltration [14], and more recently from Pandelova and colleagues [33] who indicated that Ptr Tox A elicits an ethylene response, although the production of ethylene gas *in situ* has not yet been measured. The production of aminocyclopropane-1-carboxylic (ACC) acid implies that the methyl group from Ado Met is ultimately destined for ethylene biosynthesis [34] since ACC has not been shown to be used for any other purpose in plants. It is therefore possible that Ptr ToxA triggers the production of ethylene in sensitive leaves, since ethylene is the only pathway of Ado Met with a known role in the biotic stress response [35]. Ethylene accelerates both necrosis and chlorosis associated with disease in susceptible plants [36] and it has been shown to activate ROS-generating enzymes. Hence ethylene is a good candidate for the source of ROS which appear 18 hpi. Alternatively ethylene may be working in conjunction with ROS to amplify the necrotic lesions [34]. ROS also act as a trigger for senescence in plants injured with ozone [37].

The propagation of the ROS signal may be aided by the progressive decrease of GTP-binding protein typ A, spot 8. Typ A, also known as Bip A type GTPase, plays a key role in oxidative stress tolerance in *Suaeda salsa* [38] thus its decrease in sensitive wheat leaves might cause the sensitive plant to become more susceptible to oxidative stress. Therefore, from the identified proteins of the sensitive plants along with published transcriptional data it is possible that senescence-like features are triggered in sensitive leaves, possibly by ROS produced by ethylene.

### Insensitive response

The insensitive wheat cultivar, Amazon, did not show a phenotypic difference between Ptr ToxA and buffer-infiltrated control. It has previously been assumed that the plant’s inability to recognize the toxin results in no biochemical or physiological changes in insensitive tissue. Here we demonstrate that although there are no macroscopic changes, measurable and reproducible changes do occur in the proteome.

Immediate changes in the abundance of photosynthetic protein were seen in chlorophyll A-B binding protein of LHCII type II, spot 2, which showed a relatively large decrease in expression at day 1 with relatively smaller changes as time progressed. Another chloroplastic photosynthetic protein Rieske Fe-S protein, spots 10 and 11, was found in two isoforms with differing abundance between the treatments. Spot 10 (high pI) appeared only in the control, while spot 11 (low pI) appeared only in the toxin infiltrated plant. Functionally, Rieske Fe-S is a protein required by cytochrome b_6_/f complex for structural stability [39]. Few studies have been published concerning the different isoforms, however mutants of *Chlamydomas reinharditii* that lacked functional Rieske protein completely suppressed the photosynthetic electron flow [40].

The other proteins analyzed in insensitive wheat are involved in general metabolism and protein synthesis. Glyceraldehyde 3 phosphate dehydrogenase, spot 25, a key enzyme of glycolysis showed a consistent increase and hydroxypyruvate reductase (HPR), spot 24, is an enzyme associated with the recycling of carbons in the photorespiratory cycle was highly expressed throughout [41]. Interestingly the HPR identified here shows a high degree of homology to HPR that is transcriptionally regulated by cytokinin, a known inhibitor of senescence [42].

The remaining changes are from proteins involved in translation: an increase of 5,10 methylene-tetrahydrofolate reductase, spot 4, and elongation factor 1 gamma, spot 13. Methylene-tetrahydrofolate reductase produces methionine, the first amino acid required for protein synthesis and its increase may be connected to the increased levels of elongation factor 1 gamma.

Based on this study, even though the insensitive leaves are phenotypically asymptomatic, changes are occurring in proteins related to photosynthesis and metabolism. It would be of interest to characterize the extent of the photosynthetic and metabolic changes further using more sensitive proteomics techniques, since a general increase in metabolism and sudden changes in photosynthesis are features also seen in senescence inhibition [43]. This is a possibility since the HPR identified was homologous to HPR activated by cytokinin, a senescence inhibitor. Further experiments are required to confirm this, but the preliminary evidence gathered here support this possibility.

## Conclusions

This study reveals evidence from the proteomes of wheat leaves that sensitive and insensitive cultivars may be reacting to Ptr ToxA in complementary ways. Even though the toxin is not internalized into cells of the insensitive cultivar, there are changes in the proteome, and this is the first report of such changes. Findings support the involvement of ethylene, although it was not possible to compare ethylene levels between the cultivars. The nature of the plant-pathogen interaction permits using purified toxin to mimic disease, and this both eliminates the pathogen proteome and ensures a synchronous response in inoculated leaves. Techniques which can gain deeper access into the proteome may help to elucidate the biochemical changes that are occurring in insensitive plants infiltrated with Ptr ToxA, a plant previously thought to be non-reacting.

## Methods

### Spore germination and liquid culture of *P. tritici-repentis*

*Pyrenophora tritici-repentis* (Died.) Drechsler, isolate 86–124, Race 2 which produces Ptr ToxA was obtained from Dr. L. Lamari, Department of Plant Science, University of Manitoba. Dried wheat leaves infected with isolate 86–124 were placed on V8- Potato Dextrose Agar (PDA) at 20°C, until mycelia colonies reached a 2 cm diameter. After flooding with sterile distilled water mycelia were knocked down with a glass tube and incubated for a 12 h light/dark cycle to induce sporulation. Spores were collected and approximately 2.2 x 10^5^ spores were added to eight batches of 250 ml Fries Medium (5 g ammonium tartrate, 1 g NH_4_NO_3_, 0.5 g MgSO_4,_ 1.3 g KH_2_PO_4_, 30 g sucrose, 1.0 g yeast extract, and 2.0 ml trace elements (167 mg, LiCl_3,_ 10 mg CuCl_2_, 34 mg MoO_4_, 72 mg MnCl_2_, and 80 mg CoCl_2_ in 1 L). Multiple plates of each media were prepared in square culture plates (Corning, 245 mm × 245 mm) and grown for a total of 3 weeks at 20°C in still culture. At 2 weeks, 1 ml of the culture was sampled, diluted 1:1 and infiltrated into the first and second leaves of wheat seedlings. Leaves were examined one and two days post-infiltration, and marked if a necrotic lesion formed. Lesion-forming cultures were incubated for a total of three weeks, the rest were discarded.

### Plant growth conditions and infiltration

The Tox-A sensitive and insensitive wheat (*Triticum aestivum* L*.)* cultivars used in this study were Glenlea (susceptible) and Amazon (resistant), respectively. Amazon is a near isogenic backcross derivative of Glenlea that has an introgressed *tsn 1* allele. It was created through a cross between Glenlea and Salamouni (highly resistant to Tan Spot) which was then backcrossed 5 times to Glenlea. The two cultivars were grown in a greenhouse with 16 h of light at 21°C and 8 hr of dark at 16°C. Plants were grown for two weeks for use in bioassays.

### Ptr ToxA purification

Toxin purification was performed as described by Ballance *et al*. with minor modifications [44]. Media were filtered through two layers of Whatman #1 paper and 0.45 μm nylon mesh. The filtrate was concentrated by centrifugation through 5000 molecular weight cut off (MWCO) spin columns (Vivaspin: Vivascience, UK) to one twentieth of their original volume and diluted 1 in 10 with 10 mM sodium acetate pH 5.0. The toxin was purified with two rounds of CM-cellulose ion exchange chromatography, assaying for the presence of toxin in column fractions by leaf infiltration. In addition, 50 μl of each fraction was separated by electrophoresis on a 10 cm Tris-tricine gel to evaluate the protein composition of each fraction. Active fractions were pooled. Purified active fractions were collected from the media and analyzed on a 10-20% Tris-tricine SDS polyacrylamine gradient gel to assess purity and M_r_ of the isolated protein, and desalted on Sephadex G-25 (HiPrep 26/10: GE Healthcare, Piscataway NJ) attached to a Biologic HR (BioRad Laboratories: Hercules CA) workstation. The sequence of the purified Ptr ToxA was verified by *de novo* sequencing from mass spectra. To calculate yield, the absorbance of the solution was measured at 280 nm and the protein concentration determined based on *E*_M_ = 20,800 for the 13.9 kDa Ptr ToxA protein [44]. Purified toxin was diluted to 1 μM and infiltrated into the apoplastic space of wheat leaves with a Hagborg device. Approximately 100 μl of solution was injected into a 3 cm leaf region. The infiltrated region was marked with India ink.

### Leaf protein extraction

Infiltrated regions of leaves were collected at day 1, day 2 and day 3 post infiltration and immediately frozen in liquid nitrogen. Three infiltrated frozen leaf regions (approx. 1 g) were ground to a fine powder and transferred into a 15 ml glass centrifuge tube. Protein was precipitated from this with 10 ml of −20°C chilled acetone, with 10% (w/v) TCA and 0.07% (w/v) DTT overnight at −20°C. Samples were centrifuged at 10,000 g for 20 min at 4°C and pellets washed seven times in 10 ml chilled acetone with 0.07% (w/v) DTT. The final pellet was dried under a stream of nitrogen gas and stored at −80°C.

### Two dimensional electrophoresis

Samples stored at −80°C were left to equilibrate to room temperature, and dissolved in 2 ml of isoelectrofocusing (IEF) solution (7 M urea, 2 M thiourea, 20 mM DTT, 4% (w/v) CHAPS, 0.02% (v/v) of 40% stock ampholyte (Biolyte 3–10, BioRad Laboratories). Proteins were shaken continuously for 2 h followed by sonication with a microtip sonicator, (Misonix, Farmingdale NY) for a total of 25 s, with 5 s pulses. After sonication the solution was centrifuged at 10,000 g for 20 min. The supernatant was retained and exchanged three times with IEF solution using centrifugal filter devices (2 ml 5000MWC: Vivaspin). During the final exchange, the solution was concentrated to approximately 400 μl. This was centrifuged at 80,000 g for 30 min. The protein concentration was determined using a Bradford protein assay with BSA as a standard.

Isoelectric focusing of 500 μg protein/450 μl was performed on 24 cm IPG strips (pH 4–7: GE Healthcare) exactly as described previously for a total of 58.9 kVh [45]. The second dimension was sodium dodecyl sulfate- polyacrylamide gel electrophoresis (SDS-PAGE) and was carried out in an Ettan Dalt 6 unit (GE Healthcare) according to the manufacturer’s instructions with some modifications. Polyacrylamide gels (12.5%) were electrophoresed at 22.5°C and 2 W for 1.5 h to allow for slow entry of proteins into the gel, followed by 17 W per gel until the dye front had traveled the distance of the gel. Gels were stained with Coommassie Blue R-250 overnight. Once destained, gels were scanned to produce TIFF images. Gel runs were repeated until two reproducible runs were obtained.

To obtain quantitative data each spot on the gel was measured using Quantity One (BioRad Laboratories), which assigns a value to the intensity of the spot. For each spot to be picked for analysis, an adjusted volume intensity which takes into account the variability of the background was used. These values were obtained for the control and toxin treatments in both hosts and both biological replicates. The data were compiled as follows: two biological replicates of the same spot in the same treatments were averaged and then the averaged values of the treatments, control and toxin were compared to each other as a ratio of the experimental value relative to control. A ratio >1 indicates an increased expression in treated samples, values <1 indicate a decrease in expression. Negative values, which indicated an absence of the spot in a time treatment, were arbitrarily assigned a value of 1 so that ratio could be calculated to reflect an increase or a decrease of expression as indicated above. All spots which demonstrated the same trend visually in two biological replicates were selected for analysis.

### Mass spectrometry

Proteins of interest were cut from gels in cubes of approx. 1 mm^3^. In-gel trypsin digestion and subsequent peptide extraction was carried out as described previously [41]. Mass spectra of the resulting tryptic peptides were acquired in a linear ion trap mass spectrometer (LTQ XL: Thermo Fisher, San Jose CA) as described previously [45]. Briefly, peptides were separated through a C_18_ column (12 cm fused silica column, 75 μm ID, packed with Vydac C_18_, 5 μm bead, 300 Å pores) coupled directly to the mass spectrometer via a nanoelectrospray ionization source. An acetonitrile gradient (4% (v/v) to 40% (v/v) in 1% (v/v) FA and 0.1% (v/v) acetic acid) was delivered at 250 nl/min over 30 minutes (Ultimate 3000 HPLC: Dionex, Bremen, Germany). A survey scan was acquired over the range m/z 300–2000 and was followed by five MS/MS scans of the most intense ion peaks, with dynamic exclusion for 30 s to increase coverage. Protein identification of the MS/MS spectra was performed using the Mascot search engine (Matrix Science, London UK, v2.4). The following parameters were set: a monoisotopic mass accuracy of ±1 Da; up to one missed cleavage; peptide charge of +2 or +3, a fixed modification of carbamidomethyl (Cys) and variable modifications of oxidation (Met) and deamidation of (Gln/Asn). Raw MS data files were converted to a DTA files which were used to query the NCBI non-redundant database, limited to *Viridiplantae.* Proteins were considered correctly identified if returns contained two or more peptides with a significant score as defined [46].

## Additional file

Additional file 1: Table S1. Intensities values obtained from Quantity One for proteins separated by 2-DE.

## Abbreviations

Hpi: Hours post-inoculation
Ptr ToxA: *Pyrenophora tritici-repentis* Toxin A
ROS: Reactive oxygen species
